# The antiapoptotic OPA1/Parl couple participates in mitochondrial adaptation to heat shock^☆^

**DOI:** 10.1016/j.bbabio.2012.05.001

**Published:** 2012-10

**Authors:** Luiza K. Sanjuán Szklarz, Luca Scorrano

**Affiliations:** a Dulbecco-Telethon Institute, Venetian Institute of Molecular Medicine, Via Orus 2, 35129 Padova, Italy; b Department of Cell Physiology and Medicine, University of Geneva, 1 Rue M. Servet, 1206 Geneve, Switzerland

**Keywords:** BAK, Bcl-2 associated killer, BAX, Bcl-2 associated protein X, BCL-2, B-cell lymphoma 2, DRP1, dynamin related protein 1, FCCP, cyanide m-fluorophenylhydrazone, FIS1, fission 1, HSP, heat shock protein, IMM, inner mitochondrial membrane, IMS, intermembrane space, MEFs, mouse embryonic fibroblasts, OMM, outer mitochondrial membrane, OPA1, optic atrophy 1, MFN, mitofusin, PARL, presenilin associated rhomboid like, Mitochondrion, OPA1, PARL, Heat shock response, Cytochrome *c* release, Apoptosis

## Abstract

The mitochondria-shaping protein optic atrophy 1 (OPA1) has genetically distinguishable roles in mitochondrial morphology and apoptosis. The latter depends on the presenilin associated rhomboid like (PARL) protease, essential for the accumulation of a soluble intermembrane space form of OPA1 (IMS-OPA1). Here we show that OPA1 and PARL participate in the heat shock response, a stereotypical cellular process of adaptation to thermal stress. Upon heat shock, long forms of OPA1 are lost and mitochondria fragment. However, mitochondrial fusion is dispensable to maintain viability, whereas IMS-OPA1 is required. Upon conditioning—a process of mild heat shock and recovery—IMS-OPA1 accumulates, OPA1 oligomers increase and mitochondria release less cytochrome *c*, ultimately resulting in cellular resistance to subsequent apoptotic inducers. In *Parl*^−/−^ cells accumulation of IMS-OPA1 is blunted and conditioning fails to protect from cytochrome *c* release and apoptosis. Thus, the OPA1/PARL dependent pathway of cristae remodeling is implicated in heat shock. This article is part of a Special Issue entitled: 17th European Bioenergetics Conference (EBEC 2012).

## Highlights

► Heat shock causes cleavage of the cristae regulator OPA1. ► Heat shock causes PARL-dependent accumulation of soluble antiapoptotic OPA1. ► Soluble OPA1 confers secondary resistance to apoptosis to heat shocked cells. ► OPA1 is essential also for heat shock conditioning.

## 1 Introduction

Mitochondria are versatile and dynamic organelles that play a key role in the regulation of metabolism, cellular signaling and apoptosis, during which they release cytochrome *c* and other cofactors that once in the cytosol contribute to the activation of the effector caspases required to demolish the dying cell [52]. The process of mitochondrial permeabilization is controlled by the Bcl-2 family of oncogenes: the so called BH3-only members (like BID and BIM) transduce private apoptotic signals to the organelle, activating the multidomain proapoptotic proteins of the family (that include BAX and BAK) responsible for the permeabilization of the outer mitochondrial membrane. The anti-apoptotic members like BCL-2 itself regulate this process, preventing at multiple points the activation of the proapoptotic multidomains [48].

Morphological and ultrastructural alterations accompany the recruitment of mitochondria by the cell death pathway, including fragmentation of the network [17,30] and remodeling of the cristae [43,54] in order to allow the complete release of cytochrome *c*. Mitochondrial shapes vary depending on the organism, the cellular type, the metabolic state and the environmental conditions [4]. A growing family of mitochondria-shaping proteins controls the morphology of the organelle. In mammals, Fission 1 (FIS1), mitochondrial fission factor (MFF) and dynamin related protein 1 (DRP1) regulate mitochondrial fission [24,35,44,55]; mitofusins (MFN) 1 and 2 and optic atrophy 1 (OPA1) control the fusion process [8,40,41]. Interestingly, OPA1 has genetically distinguishable functions in mitochondrial fusion and release of cytochrome *c* during apoptosis [18]. The function of OPA1 is tightly controlled at the genetic and post-translational level: OPA1 gene undergoes alternative splicing and the protein is proteolyzed, leading to the generation of several forms with different electrophoretic mobilities. Under normal conditions, in most tissues 2 long and 3 short forms of the protein can be distinguished; both long and short OPA1 are required to maintain mitochondrial fusion [46]. Several proteases have been found to be involved in the generation of the short forms of OPA1, including the matrix AAA protease paraplegin and AFGL3 and the intermembrane space AAA protease YME1 [16,21,23]. Following mitochondrial dysfunction, an additional cleavage by the ATP independent protease OMA1 inactivates the long forms of OPA1 leading to an accumulation of short forms of OPA1 [16] and to segregation of fragmented mitochondria from the network [14]. In addition, the short forms of OPA1 constitutively produced by the AAA proteases seem also to be the substrate of a mitochondrial rhomboid protease called presenilin associated rhomboid like (PARL). PARL was originally discovered in a yeast two hybrid screening for presenilin interactors. It then turned out to be a mitochondrial enzyme that in yeast (where it is christened Pcp1p) and in *Drosophila melanogaster* cleaves the orthologs of OPA1 [31,32]. Considerable confusion has emerged on the role of PARL, based on our early report that it is required for the accumulation of a soluble form of the OPA1, essential for apoptosis but not for mitochondrial fusion [9]. This report ingenerated the idea that the generation of the short forms of OPA1 depended on PARL (see for example the introduction in [15,29]). Conversely, we ourselves introduced the possibility that in analogy with other intramembrane proteolytic cascades such as that of Notch [53], PARL acts downstream of other protease(s) [9]; despite our words of caution, the dependence of the accumulation of the soluble form of OPA1 on PARL has been equaled to a broader role for the protease in the constitutive generation of the short forms of OPA1. In conclusion, our current understanding of OPA1 cleavage is certainly increasing, yet several areas remain obscure: for example, it is still largely unknown how the activity of the different proteases is controlled; whether they operate in parallel or in series (with the remarkable exception of Parl that seems to operate only on the lower MW forms of OPA1); which are the domains implicated in substrate recognition by the proteases, as well as their exact cleavage site in OPA1. Altogether, these black boxes bamboozle our interpretation of how these proteases participate in the regulation of mitochondrial morphology and apoptosis. In particular, in the case of Parl it is unclear if the proposed role in apoptosis mediated by OPA1 can be extended to stimuli other than drugs activating the intrinsic pathway of cell death; and whether it participates in cellular adaptation.

When cells are exposed to stressful stimuli that results in the inhibition of protein synthesis, such as mRNA translation inhibitors and UV irradiation, mitochondria undergo hyperfusion [50]. During starvation a similar process of mitochondrial elongation occurs, and it is mirrored at the ultrastructural level by an increase in the surface of the cristae where the ATP synthase oligomerizes to maximize its efficiency; and depends on a signaling cascade triggered by a rise in cyclic AMP levels and impinging on Drp1 to cause unopposed mitochondrial fusion [20]. Exposure to stressful conditions results in different responses at the cellular level that stereotypically follow a scheme where the attempt of compensation precedes suicide of the stressed cell. This paradigm is best evident in the case of ER stress, when the activation of the unfolded protein response first tries to compensate the organellar damage, then signals the activation of the apoptotic pathway. Mitochondrial elongation during starvation (and other stressful conditions) seems to be another example of this paradigm of compensation/suicide, where changes in the shape of the organelle regulate the continuum between survival and death of the cell.

In addition to nutrient availability, cells can respond efficiently also to changes in temperature by activating the so called heat shock response. In response to thermal stress, cells synthesize and accumulate heat shock proteins (HSPs), chaperones or proteases that protect the cell in response to potentially damaging conditions [38]. Of note, chaperones are key in the mitochondrial import of proteins, highlighting a cross-talk between thermal adaptation and the organelle [45]. In addition, if the temperature surpasses a duration or intensity threshold, the cell is committed to death, via activation of the mitochondrial pathway of apoptosis [38]. HSP 27 and 70, two main players in the heat shock response, block apoptosis also at a post-mitochondrial level, by interacting with cytochrome *c* and with the other released cofactor AIF in the cytosol [5,39].

One of the most remarkable aspects of the heat shock response is that upon transient exposure of cells to mild heat stress, it renders cells resistant to further cellular insults. This might be linked to the ability of overexpressed HSPs to block apoptosis. HSP27 and HSP70, two main heat shock response proteins, prevent apoptosis at a post-mitochondrial level, by interacting with cytochrome *c* and AIF cofactor released in the cytosol [5,38]. HSPs might prevent apoptosis not only by interfering with the activation of the apoptosome, but also by interfering with key molecules of the extrinsic pathway such as DAXX and JNK [38]. Whether HSPs can also interfere with mitochondrial permeabilization and release of apoptotic cofactors is still a matter of debate; in particular, whether mitochondrial changes induced by heat stress directly participate in the acquisition of cellular resistance against apoptotic stimuli remains unclear [3].

Here we analyzed whether OPA1 participates in the heat shock response. We show that upon heat shock the antiapoptotic soluble form of OPA1 accumulates in mitochondria and that this accumulation favors heat stress-induced tolerance against subsequent apoptotic cell death. A genetic analysis proved that the rhomboid protease PARL participates in cellular adaptation to heat shock by impinging on soluble OPA1. Thus, our data suggest a role for OPA1 in heat stress-induced cytoprotection against apoptotic stimuli.

## 2 Methods

### 2.1 Cell culture

Mouse embryonic fibroblasts (MEFs) of the indicated genotype were cultured at 37 °C in 5% CO_2_ as previously described [20]. For heat shock treatment, cells were brought to a confluence of around 85% at 37 °C and then cultures in a 5% CO_2_ incubator at 42 °C (for mild heat stress) or at 45 °C (for severe heat stress) for the time indicated. For conditioning, cells were exposed to mild heat shock followed by recovery at 37 °C.

### 2.2 Mitochondrial morphology analysis

8 × 10^4^ MEFs were seeded in plastic dishes containing a glass coverslip. After 24 h cells were transfected with mitochondrial yellow fluorescent protein (mtYFP) for imaging of live cells or immunostained with a FITC-conjugated anti TOM20 antibody. Heat shock treatment of cells started 20 h post-transfection. Images of mtYFP or FITC were acquired using a Leica TCS SP5 inverted confocal microscope equipped with a 63 ×, 1.3 NA PLANApo objective (Leica) using the appropriate excitation lasers and emission filters, as previously described [20]. Morphometric analysis was performed as previously described [8].

### 2.3 Cell viability assays

1.2 × 10^5^ MEF cells were seeded in 12 well plastic plates, after 24 h cells were exposed to heat shock treatment for the indicated time points (42 °C for mild heat shock; 45 °C for severe heat shock) and cell viability was determined by flow cytometry. For co-transfection experiments, 7 × 10^4^ MEFs were seeded on plastic 12-well plates, after 18 h of growth cells were co-transfected with Transfectin (Biorad) and 24 h post transfection cell viability was determined by flow cytometry. Cells were harvested and stained with propidium iodide (PI) and Annexin-V-FITC (Bender) according to manufacturer's protocol. Flow cytometry analyses were performed using a FACS Calibur cytometer (Becton-Dickinson). Viability was measured as the percentage Annexin-V, PI negative cells.

### 2.4 Mitochondrial biochemistry

Mitochondria were isolated from MEFs by differential centrifugation as described previously [19]. For sub-fractionation of mitochondria, isolated mitochondria were hypotonically swollen in 10 mM KPi (pH 7.4), centrifuged for 10 min at 12,000 ×*g* and the pellet fraction was washed with 150 mM KCl, 10 mM KPi (pH 7.4). Proteins in the pooled supernatant fractions were precipitated with 10% TCA. Soluble and membrane associated mitochondrial proteins were contained in the supernatant fraction and the pellet fraction contained membrane integrated proteins. Samples were solubilized in loading buffer, resolved by SDS-PAGE and analyzed by Western blot.

Cytochrome *c* release and BAK crosslinking in response to recombinant cBid were measured as described previously [42].

## 3 Results

### 3.1 Reversible fragmentation of the mitochondrial network in mouse embryonic fibroblasts (MEFs) exposed to heat shock

**Fig. 1:**
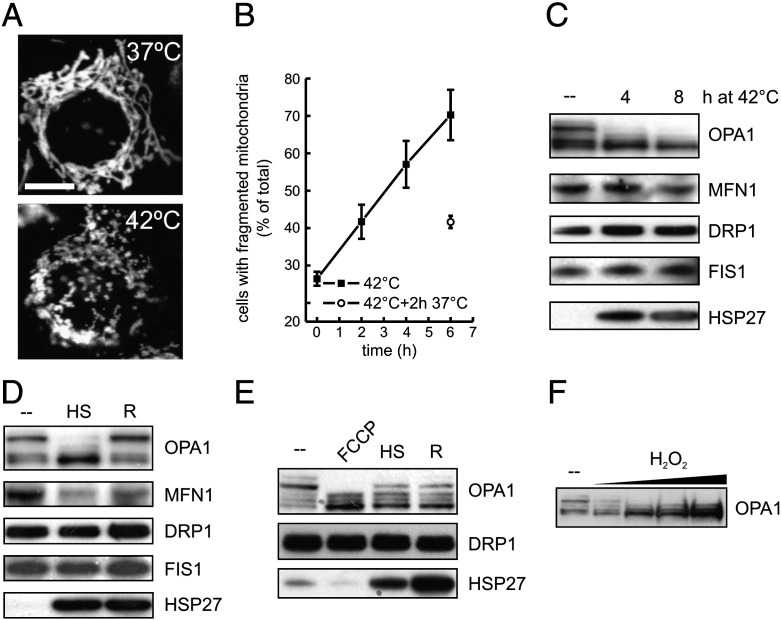
Mild heat shock causes changes in OPA1 electrophoretic mobility and induces mitochondrial fragmentation. (A) Representative confocal images of wild type MEFs grown at 37 °C or exposed to the indicated temperature for 4 h, fixed and immunostained against the mitochondrial marker TOM20. (B) Morphometric analysis of mitochondrial shape upon heat shock. Cells expressing mtYFP were exposed to the indicated temperatures for the indicated times and confocal images were acquired. Three hundred random images of mtYFP fluorescence were acquired, stored and classified. Data represent ± SE of 3 independent experiments. (C–D) Equal amounts (25 μg) of proteins from wild type MEFs grown at 42 °C for the indicated times (C) or exposed to heat shock (HS, 42 °C for 3 h) or to heat shock followed by recovery (R, 37 °C for 6 h) (D) were analyzed by SDS-PAGE and immunoblotting using the indicated antibodies. (E–F) Equal amounts (25 μg) of proteins from wild type MEFs treated where indicated with 2 μM FCCP, subjected to heat shock (HS, 42 °C for 6 h) or conditioning (R, 42 °C for 3 h followed by 6 h at 37 °C) or (E) treated with increasing (0.1, 0.25, 0.5 and 1 mM) concentrations of H_2_O_2_ were separated by SDS-PAGE and immunoblotted using the indicated antibodies.

In order to address if changes in mitochondrial shape participate in the cellular response to heat shock we first decided to inspect the morphology of the mitochondrial network after exposure of cells to heat stress. Following exposure of wild type MEFs to mild heat shock (42 °C), confocal microscopy of the outer mitochondrial membrane protein TOM20 in fixed cells and of mitochondrial YFP in live cells (not shown) showed consistent mitochondrial network fragmentation whose extent was dependent on the time of heat treatment (Fig. 1A and B). Interestingly, fragmentation was reversible, as indicated by the recovery of elongated morphology upon return of the cells to normal growth temperature (Fig. 1B). To understand the molecular mechanism behind mitochondrial fragmentation induced by heat shock, we compared by immunoblotting the levels of mitochondrial shaping proteins in wild type MEFs grown at normal temperature with those retrieved in MEFs exposed to mild heat shock. Efficient induction of the heat shock response, as indicated by the upregulation of the cytosolic chaperone Hsp27 [5] did not result in an increase in the mitochondrial fission proteins, FIS1 and DRP1 or of the mitochondrial fusion protein MFN1 (Fig. 1C). Marked differences were observed however upon inspection of the levels and electrophoretic pattern of the mitochondrial fusion protein OPA1. As a consequence of the alternative splicing of the *Opa1* gene, and of proteolytic processing of the OPA1 protein, two long and three short forms of OPA1 can be retrieved upon immunoblotting of MEFs lysates [1] (Fig. 1C). Following heat treatment, short forms of OPA1 accumulated, concomitant with the disappearance of the long ones (Fig. 1C). These changes in the pattern of OPA1 electrophoretic mobility were reversible when cells returned to normal growth temperature (Fig. 1D and E) and were clearly different from the accumulation of cleaved products observed upon mitochondrial depolarization induced by the uncoupler FCCP (Fig. 1E) or after mitochondrial damage by increasing concentrations of the oxidant hydrogen peroxide (Fig. 1F) [14]. Indeed, we could not appreciate any loss in mitochondrial membrane potential mild heat shock, as measured by the potentiometric dye tetramethyl rhodamine methyl ester (TMRM). In conclusion, our experiments indicate that mitochondrial morphology is reversibly modulated during mild heat shock and this correlates with changes in the forms of OPA1, with unbalance between long and short forms of the protein that are both required for efficient mitochondrial fusion [13,47].

### 3.2 Intermembrane space, soluble OPA1 is required to protect cells from death by heat shock

**Fig. 2:**
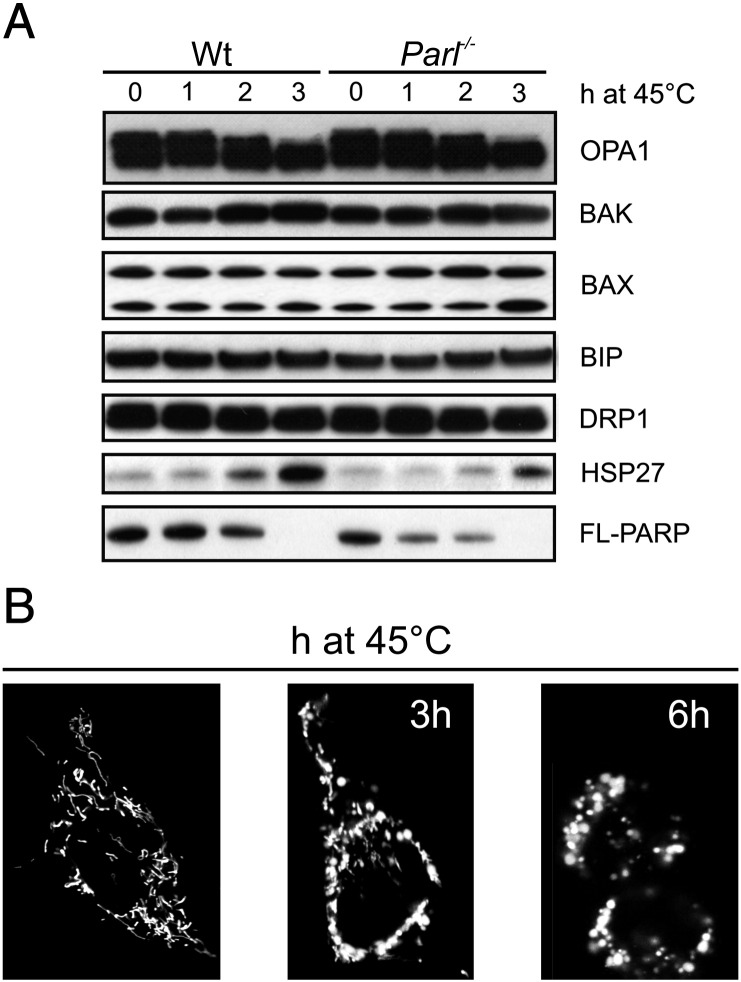
Severe heat stress causes mitochondrial fragmentation and OPA1 cleavage. (A) Equal amounts (25 μg) of proteins from MEFs of the indicated genotype grown at 45 °C for the indicated times were separated by SDS-PAGE and immunoblotted using the indicated antibodies. (B) Representative confocal images of wild type MEFs transfected with mtYFP and after 24 h grown at 45 °C for the indicated time.

**Fig. 3:**
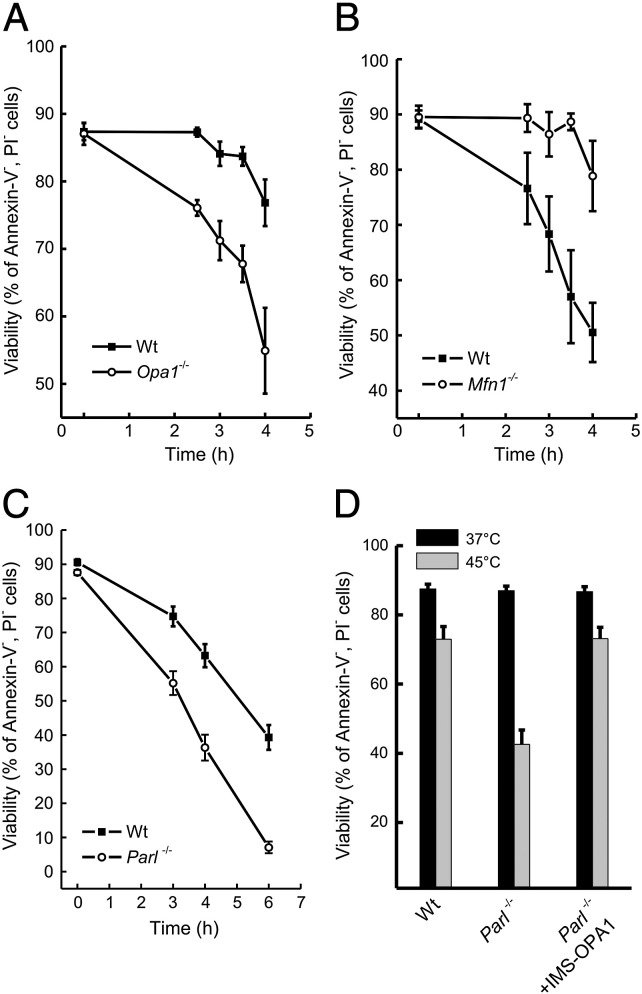
Lack of OPA1 and PARL increases sensitivity to severe heat shock. (A–C) MEFs of the indicated genotype were exposed to heat shock (45 °C) and at the indicated times viability was determined cytofluorometrically as the percent of Annexin-V-Fluos, PI negative cells. (D) MEFs of the indicated genotypes were co-transfected with GFP and the indicated plasmids and exposed to severe heat shock. At the indicated times viability was determined cytofluorometrically as the percent of GFP-possitive, Annexin-V-Alexa568 negative cells.

OPA1 functions in mitochondrial morphology and in apoptosis can be genetically distinguished: while fusion driven by OPA1 requires the outer membrane mitofusin 1 [8], regulation of cristae remodeling and cytochrome *c* release depends on PARL [9,18], a rhomboid protease of the inner mitochondrial membrane [25,36]. We therefore wondered whether the changes in OPA1 forms upon heat shock not only caused mitochondrial fragmentation, but it also impacted on cell death. When we compared viability of wild type (wt) and *Opa1*^*−/−*^ MEFs exposed to mild heat shock (42 °C), we could not detect any death for up to 8 h (not shown). We therefore decided to turn to severe heat shock (45 °C), upon which we could also observe an increase in the levels of HSP27, cleavage of OPA1 and at later time points cleavage of the caspase substrate PARP [11], as testified by the disappearance of its long form, in both wt and *Parl*^*−/−*^ MEFs (Fig. 2A). A more detailed analysis of the forms of OPA1 that were lost upon severe heat shock revealed that the long forms were lost as early as after 60 min after heat shock in both wt and *Parl*^*−/−*^ MEFs (not shown). These changes were accompanied by mitochondrial fragmentation and aggregation, as testified by the confocal imaging of the mitochondrial network labeled with a mitochondrially targeted YFP (Fig. 2B). *Opa1*^*−/−*^ cells resulted more sensitive than their corresponding wild type counterparts to severe heat shock (Fig. 3A). To address if the observed sensitivity to heat stress was a mere consequence of the increased mitochondrial fragmentation of *Opa1*^*−/−*^ MEFs [45,46], we measured the sensitivity to heat shock of *Mfn1*^*−/−*^ MEFs where mitochondria are also fragmented [7,8]. As opposed to *Opa1*^*−/−*^ MEFs, *Mfn1*^*−/−*^ cells were even less sensitive than their wt counterpart to heat treatment (Fig. 3B), indicating that a fragmented mitochondrial network does not per se sensitize to heat shock. We therefore tested whether the increased sensitivity to cell death upon severe heat of the *Opa1*^−/−^ MEFs was related to the antiapoptotic function of OPA1, that resides in the small soluble form of the protein. To this end we turned to *Parl*^*−/−*^ MEFs that fully recapitulated the enhanced sensitivity to heat stress of the *Opa1*^*−/−*^ MEFs (Fig. 3C). To verify if the enhanced sensitivity to heat treatment of *Parl*^*−/−*^ MEFs was a consequence of the reduced production of soluble OPA1, we complemented them with the soluble form of OPA1 (IMS-OPA1) [23]. IMS-OPA1 fully corrected the increased sensitivity to cell death induced by heat shock of *Parl*^*−/−*^ MEFs (Fig. 3D). Our data indicate that upon severe heat shock, short, soluble OPA1 plays a crucial role in the protection from cell death, suggesting a role for the OPA1/PARL antiapoptotic couple also in this form of cell death.

### 3.3 Heat shock induces the PARL dependent accumulation of soluble OPA1

**Fig. 4:**
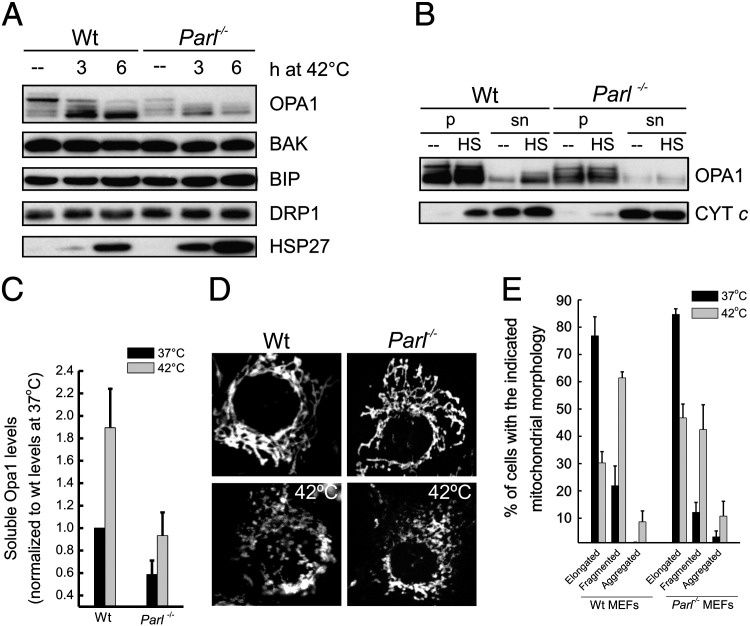
Accumulation of soluble OPA1 upon mild heat shock depends on PARL. (A) Equal amounts (25 μg) of proteins from MEFs of the indicated genotype exposed to mild heat stress (42 °C) for the indicated time points were analyzed by SDS-PAGE and immunoblotting using the indicated antibodies. (B) Equal amounts of mitochondria (50 μg) isolated from cells of the indicated genotype, grown at 37 °C (−−) or at 42 °C (HS) for 4 h, were fractionated into a soluble (sn) and a membrane integral protein fraction (p). The obtained fractions were analyzed by SDS-PAGE and immunoblotting using the indicated antibodies. (C) Densitometric analysis of levels of soluble OPA1. Experiments were performed as in (B). Data represent average ± SE of 4 independent experiments. (D) Representative confocal images of MEFs of the indicated genotype immunostained for mitochondrial TOM20. Where indicated, cells were grown at 42 °C for 4 h. (E) Morphometric analysis of mitochondrial shape upon heat shock. Experiments are as in D. Three hundred random images of TOM20 fluorescence were acquired, stored and classified. Data represent ± SE of 3 independent experiments.

We next asked whether during thermal stress the soluble form of OPA1 accumulates in a PARL dependent fashion. First, we noticed that upon mild heat shock (42 °C) the lack of PARL did not affect the heat shock response, as judged by the efficient accumulation of HSP27. In addition, cleavage to lower molecular weight forms of OPA1 was as efficient as in wild type cells (Fig. 4A). We then followed the accumulation of soluble OPA1 in the mitochondrial intermembrane space [9]. After exposure of MEFs to heat shock, the soluble form of OPA1 accumulated in wt but not in *Parl*^*−/−*^ MEFs (Fig. 4B and quantification in C). Lower levels of the endogenous soluble form of OPA1 in *Parl*^*−/−*^ mitochondria do not result from overall reduced levels of endogenous mitochondrial proteins, since levels of other mitochondrial protein markers were comparable in wild type and *Parl*^*−/−*^ mitochondria (Fig. 4A). The reduced production of soluble OPA1 in *Parl*^*−/−*^ MEFs was not associated with increased fragmentation, as evidenced by confocal microscopy showing reversible fragmentation of mitochondrial network also in *Parl*^*−/−*^ MEFs (Fig. 4D and quantification in E). On the other hand, *Parl*^*−/−*^ MEFs were more sensitive to heat shock, as indicated by their increased death after exposure to severe heat stress (45 °C, see Fig. 3). In conclusion, during heat shock cells accumulate the soluble form of OPA1 in mitochondria and this correlates with enhanced resistance to cell death.

### 3.4 PARL is required for conditioning upon heat shock

**Fig. 5:**
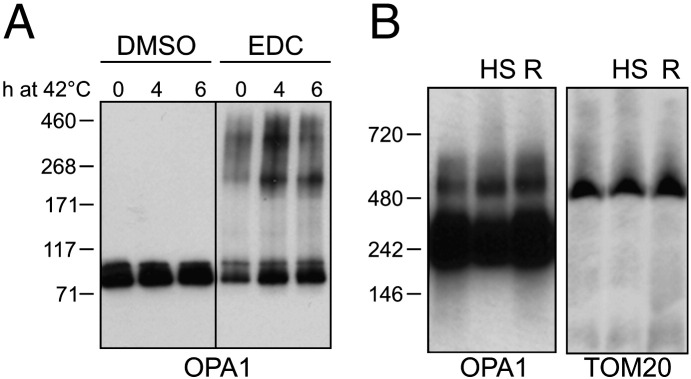
Increased levels of OPA1 oligomers upon mild heat shock. (A) Equal amounts of mitochondria isolated from wt MEFs grown at 42 °C for the indicated time points were treated as indicated, solubilized in loading buffer, analyzed by SDS-PAGE and immunoblotted using an anti OPA1 antibody. (B) Equal amounts of mitochondria isolated from wt MEFs grown at 42 °C for 4 h (HS) or at 42 °C for 4 h followed by 3 h at 37 °C (R) were solubilized, separated by BN-PAGE and immunoblotted using the indicated antibodies.

**Fig. 6:**
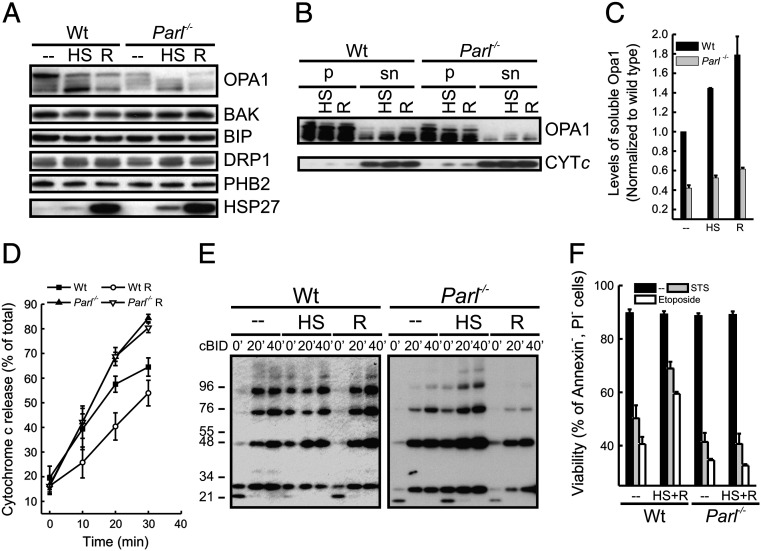
Heat shock mediated conditioning requires PARL. (A) Equal amounts (25 μg) of proteins from MEFs of the indicated genotype exposed to mild heat stress (HS; 42 °C 4 h) or to conditioning treatment (R; 3 h at 42 °C followed by 6 h at 37 °C) were analyzed by SDS-PAGE and immunoblotted using the indicated antibodies. (B) Equal amount of mitochondria (50 μg) from control, heat shocked (HS) and conditioned (R) cells were fractionated into a soluble and membrane associated protein fraction (sn) and a membrane integral protein fraction (p). The obtained fractions were analyzed by SDS-PAGE and immunoblotted using the indicated antibodies. (C) Densitometric analysis of levels of soluble OPA1. Experiments were performed as in (B). Data represent average ± SE of 3 independent experiments. (D) Mitochondria were isolated from MEFs of the indicated genotype grown at 37 °C or conditioned (R, 3 h at 42 °C followed by 6 h at 37 °C). Equal amounts of isolated mitochondria (0.5 mg/ml) were treated with 30 pmol/mg cBID for the indicated times and the amount of cytochrome *c* present in the supernatant and pellet fraction was determined. Data represent ± SE of four independent experiments. (E) Mitochondria were isolated from MEFs of the indicated genotype grown at 37 °C (−−), subjected to heat shock (HS, 3 h at 42 °C) or conditioned (R, 3 h at 42 °C followed by 6 h at 37 °C). Equal amounts of isolated mitochondria (0.5 mg/ml) were treated with 30 pmol/mg cBID for the indicated times, crosslinked using 1 mM BMH, dissolved in loading buffer, separated by SDS-PAGE and immunoblotted using the indicated antibody. (F) MEFs of the indicated genotype were conditioned (3 h at 42 °C followed by 6 h at 37 °C) and then treated for 6 h with staurosporine (2 μM) or for 48 h with etoposide (2 μM). Cell viability was measured by flow cytometry as a percent of Annexin/PI negative staining of cells.

The accumulation of soluble OPA1 during thermal stress could result in the enhanced formation of OPA1 oligomers that regulate cristae junctions and cytochrome *c* release [18,54]. We wondered whether heat-shock induced accumulation of soluble OPA1 might participate in the process of cellular conditioning, i.e. the acquisition of cellular resistance to subsequent damaging insults obtained by the exposure of cells to mild heat shock followed by their return to growth at normal temperature [38]. Therefore, we first verified the status of the OPA1 oligomers in mitochondria from cells exposed to mild heat shock as well as to thermal conditioning. Levels of OPA1 oligomers detected by cross-linking (Fig. 5A) and by blue native PAGE (Fig. 5B) were increased in mitochondria after exposure of cells to thermal stress as well as to thermal conditioning. To address if this increase in OPA1 oligomers could correlate with enhanced cellular resistance to apoptotic cell death, we turned again to wild type and *Parl*^*−/−*^ MEFs where we could observe that upon heat shock and thermal conditioning, accumulation of lower molecular weight forms of OPA1 was delayed in *Parl*^*−/−*^ MEFs (Fig. 6A). Interestingly, in wild type cells thermal conditioning induced higher accumulation of soluble OPA1 than heat shock alone, whereas soluble OPA1 was almost undetectable in conditioned *Parl*^*−/−*^ MEFs (Fig. 6B and quantification in C), at least in the time frame studied. We were therefore in a position to genetically test the role of this soluble OPA1 in the process of conditioning. Generation of soluble OPA1 by PARL participates in the control of cytochrome *c* release during apoptosis [9,18]. We therefore compared the rate of cytochrome *c* release in response to recombinant caspase 8 cleaved BID (cBID) in mitochondria purified from conditioned wild type and *Parl*^*−/−*^ MEFs. Thermal conditioning of cells leads to a reduced release of cytochrome *c* from wild type but not from *Parl*^*−/−*^ mitochondria (Fig. 6D). This difference did not result from an impaired activation of multidomain proapoptotic proteins, as testified by the similar rate of Bak activation upon cBID treatment (Fig. 6E). Thus, PARL is required for the protection against cytochrome *c* release observed in mitochondria isolated from cells exposed to mild heat shock. When we compared the rate of cell death induced by secondary apoptotic stimuli in wild type and *Parl*^*−/−*^ MEFs, we noticed that PARL was required also in situ for the heat stress induced acquisition of cellular tolerance to intrinsic apoptotic stimuli such as staurosporine and etoposide (Fig. 6F). Altogether our results indicate that, the delayed release of cytochrome *c* upon apoptotic stimuli and the enhanced resistance to apoptotic death of wild type cells conditioned with thermal stress correlate with the ability of the organelle to accumulate soluble OPA1, that participates in the control of cristae remodeling and cytochrome *c* release.

## 4 Discussion

In this study we show a role for OPA1 and PARL in the regulation of the response of the cells to heat shock. Several lines of evidence indicate that production of soluble OPA1 by PARL is required to protect cells from severe thermal stress and to render them resistant to a secondary apoptotic insult: (i) during thermal stress mitochondria fragment as a consequence of OPA1 cleavage; (ii) cells lacking *Opa1* and *Parl*, but not *Mfn1*, are more susceptible to thermal stress; (iii) *Parl*^−/−^ cells do not accumulate soluble OPA1 during heat shock and a soluble OPA1 can complement their death defect; (iv) upon conditioning by mild heat shock, the accumulation of soluble OPA1 results in increased OPA1 oligomerization, resistance to cytochrome *c* release and to secondary apoptosis that are all abolished in cells lacking *Parl*. Thus, our data extend the role of mitochondrial morphology to the regulation of heat shock response and indicate that this depends on the antiapoptotic function of the OPA1–PARL couple.

OPA1, mutated in dominant optic atrophy [2,12], is a multifaceted mitochondria-shaping protein that has genetically distinguishable functions in apoptosis [9,18,28,34] and in mitochondrial fusion [8]. The complexity of its function is reflected by the existence of 8 alternative splice variants [1] and of several proteases that participate in its post-translational processing [49]. OPA1 seems to undergo at least three types of proteolyses after removal of the mitochondrial import sequence: a first one that generates the so called “short OPA1” from the protein once it has been imported into mitochondria, that depends on AAA proteases of the inner membrane or of the matrix [16,21,23,47]; a second type that is induced in stress conditions and depends on the metalloprotease Oma1 [16,29]; and a third cleavage that represents the evolutionary remnant of the cleavage of Mgm1p, the yeast homolog of OPA1. Production of short Mgm1p in *Saccharomyces cerevisiae* depends on the rhomboid protease Pcp1p [32]; however, as mentioned above, OPA1 is processed by other proteases and PARL, the mammalian homolog of Pcp1p, intervenes on the already short form of the protein to stabilize a minor, soluble form that is required for the antiapoptotic function of OPA1 [9]. This original discovery has been interpreted as if PARL was responsible for the production of short OPA1. However, it appears clear from our previous study that PARL participates in the production of soluble, rather than short OPA1; and that the soluble OPA1 is generated from its short, already processed form [9,37]. Interestingly, levels of OPA1 seem to be variable in *Parl*^−/−^ MEFs (see for example Figs. 4 and 6). We had previously appreciated this variability and underwent a quantitative analysis of total OPA1 levels in cells where *Parl* had been ablated, without however finding any statistically significant difference [9,18]. Moreover, the enhanced apoptosis of *Parl*^−/−^ MEFs can be corrected only by IMS-OPA1 and not by the overexpression of OPA1 per se, reinforcing the concept that levels of OPA1 are not crucial for the apoptotic defect observed in the absence of *Parl*. Our current study lends further support to the notion that accumulation of IMS-OPA1 requires PARL and that this soluble form of the protein participates in the regulation of apoptosis.

Changes in the electrophoretic mobility of OPA1 are not exclusively found in response to heat stress: rapidly upon exposure of cells to multiple stimuli, OPA1 forms change to mediate mitochondrial elongation [50]; chronic inhibition of mitochondrial fission also results in changes in OPA1 forms [33], a possible explanation for the observed increase in cristae surface during the inhibition of DRP1 upon autophagy [20]. In response to mitochondrial dysfunction, OPA1 is proteolyzed to segregate dysfunctional organelles, impairing their fusion with the remaining chondriome [51]. These changes are however different from what we observed during heat shock, and most importantly during conditioning (exposure to mild heat shock followed by recovery), when OPA1 cleavage is followed by PARL mediated accumulation of soluble OPA1. Our data indicate that this complex process of multiple cleavage results in the accumulation of OPA1 oligomer and in the inhibition of cytochrome *c* release and cell death.

Conditioning by thermal stress has been extensively studied and several reports indicate that the apoptotic resistance gained by conditioned cells occurs at a post-mitochondrial level [5] or by blocking the activation of the core mitochondrial pathway controlled by BCL-2 family members [22]. Here we identify an intrinsic mitochondrial resistance mechanism that relies on the control of cytochrome *c* release by OPA1, independently of BCL-2 family members whose activation is not affected in the mitochondria isolated from conditioned cells.

A key question is whether the regulation of OPA1 during heat shock occurs only at the posttranslational level, or it is also transcriptional. When we measured total mRNA levels of OPA1 after mild heat shock we could not detect any increase (if anything, OPA1 levels seemed to be decreased, not shown). It is therefore unlikely that OPA1 is a heat shock protein in a classic way, i.e. whose levels are regulated by heat shock response elements [38]. More likely, its participation to adaptation to thermal stress fully depends on posttranslational modifications that include at least two cleavages, one by a yet unidentified protease that accumulates short OPA1, the other by PARL that produces soluble OPA1. Noteworthy, when cells are returned to normal temperature, the soluble OPA1 is already produced and accumulated in the IMS, leading to the increased levels of OPA1 oligomer. In this respect, it would be interesting to verify if the upstream cleavage of OPA1 is required for the generation of soluble OPA1 and for the stabilization of the oligomer.

The mitochondrial function of PARL has been a matter of intense debate. While mice lacking this protease are affected by multisystemic apoptosis and *Parl*^−/−^ cells can be complemented by soluble OPA1 [9], as we mentioned above the consensus that PARL acts via OPA1 is far from being reached. Other PARL substrates have been identified in the Parkinson-associated kinase PINK1 [27] and in the mitochondrial protease HTRA2 [6], albeit in this latter case following studies have excluded this possibility [26]. However, whether the reported in vivo activities of PARL that include the regulation of mitochondrial metabolism [10] depend on OPA1 or on PINK1 is unclear and will require the generation of mouse models where PARL, OPA1 and PINK1 are deleted. The data presented here reinforce the possibility that OPA1 and PARL are part of the same pathway controlling cristae remodeling, cytochrome *c* release and apoptosis.

In summary, our data identify a role for the antiapoptotic OPA1/PARL couple in the regulation of the response to thermal stress, broadening our knowledge of the processes regulated by mitochondria-shaping proteins.

## Authors' contributions

LKSS and LS conceived the research, analyzed the data and wrote the manuscript; LKSS performed the experiments.
